# Resolvin D1 Protects Against Ischemia/Reperfusion-Induced Acute Kidney Injury by Increasing Treg Percentages via the ALX/FPR2 Pathway

**DOI:** 10.3389/fphys.2020.00285

**Published:** 2020-04-03

**Authors:** Hong Luan, Chuanxiao Wang, Jianping Sun, Long Zhao, Lin Li, Bin Zhou, Shihong Shao, Xuefei Shen, Yan Xu

**Affiliations:** ^1^Department of Nephrology, The Affiliated Hospital of Qingdao University, Qingdao, China; ^2^Department of Thoracic Surgery, Qingdao Municipal Hospital, School of Medicine, Qingdao University, Qingdao, China; ^3^Department of Pathology, The Affiliated Hospital of Qingdao University, Qingdao, China

**Keywords:** acute kidney injury, Resolvin D1, regulatory T cells, ischemia-reperfusion injury, LXA4 receptor

## Abstract

**Aims:**

To evaluate whether Resolvin D1 attenuates ischemia/reperfusion-induced (IRI) acute kidney injury (AKI) via affecting Tregs.

**Materials and Methods:**

The IRI-AKI mouse model was established, and RvD1 was injected into the mouse tail vein. Further, the renal function, histological changes, injury markers and serum cytokines were detected at 24 and 72 h after IRI. Flow cytometry was used to categorize regulatory T cells (Tregs) in the spleen and kidney. Treg cells were stripped with the anti-CD25 antibody blocker PC61 to assess its role in the protective effect of RvD1 on IRI mice. CD4^+^ T cells were obtained from spleen monocytes by magnetic bead sorting and differentiated into induced Treg (iTreg) cells. The effect of RvD1 on iTreg cell differentiation was observed *in vitro*. In addition, neutralizing antibodies against the orphan receptor G-protein-coupled receptor 32 (anti-GPR32) and LXA4 receptor (anti-ALX/FPR2), both RvD1 receptor blockers, were used to evaluate the effect of RvD1 on iTreg cell differentiation. Boc-1, an ALX/FPR2 receptor inhibitor, was administered via the tail vein to observe its effects on the ameliorative efficacy of RvD1 in IRI-AKI mice *in vivo*.

**Results:**

*In vivo*, RvD1 increased Treg percentages, alleviated renal tubular injury and reduced the serum levels of IFN-γ, TNF-α and IL-6 in IRI-AKI mice, while PC61 depleted the number of Tregs and reversed the protective effects of RvD1. *In vitro*, RvD1 induced the generation of iTregs. Importantly, preincubation with anti-ALX/FPR2 neutralizing antibodies but not with anti-GPR32 neutralizing antibodies, abrogated the enhancement activity of RvD1 on iTregs. In addition, *in vivo* blockade of the receptor ALX/FPR2 by Boc-1 reversed the beneficial effects of RvD1 on the splenic and kidney Treg percentages, renal tubular injury and serum IFN-γ, TNF-α, and IL-6 levels.

**Conclusion:**

Our study demonstrates that RvD1 protects against IRI-AKI by increasing the percentages of Tregs via the ALX/FPR2 pathway.

## Introduction

Ischemia/reperfusion injury (IRI) is the primary cause of acute kidney injury (AKI), occurs in major operations (Mehta et al., 2015). Numerous IRI animal models and human histopathological studies have shown that the inflammatory response mediated by innate and adaptive immunity is an important pathophysiological change in ischemic AKI. Th1, Th2, and Th17 cells and regulatory T cells (Tregs), which are all CD4^+^ T lymphocyte subsets, act as a bridge between innate and adaptive immunity and participate in the pathological process of ischemic AKI.

Resolvins are a new family of endogenous lipid mediators that are derived from docosahexaenoic acid (DHA) and eicosapentaenoic acid (EPA) (Serhan et al., 2002). Resolvins include the D series (RvD) and E series and can alleviate inflammation (Weylandt et al., 2012). Resolvin D1 (RvD1) is biosynthesized from ω-3 DHA, and its effect is dependent on the LXA4 receptor (ALX/FPR2) and orphan receptor G-protein-coupled receptor 32 (GPR32) (Krishnamoorthy et al., 2010). RvD1 can promote neutrophil migration and enhance macrophage phagocytosis in an ALX/FPR2-dependent manner, which contributes to the resolution of inflammation (Hong et al., 2003; Sun et al., 2007; Spite et al., 2009). RvD1 improves the cardiorenal microenvironment to clear myocardial infarction-induced inflammation by increasing neutrophil and macrophages numbers and facilitates renoprotective mechanisms to limit cardiorenal syndrome (Halade et al., 2018).

Recently, RvDs were found to be effective in IRI-AKI and to function by reducing leukocyte influx and prohibiting postischemic kidney fibrosis (Duffield et al., 2006). Chen et al. (2014) also demonstrated that aspirin-triggered RvD1 is a potent anti-inflammatory mediator in lipopolysaccharide-induced AKI. Furthermore, a recent study reported that RvD1 can also modulate adaptive immunity, including affecting the balance between pathogenic Th1/Th17 cells and tolerogenic Tregs (Chiurchiu et al., 2016). Tregs are commonly known to play critical roles in controlling inflammation and maintaining immunological tolerance in various immune disease models. According to three recent studies, Tregs suppress innate immunity in the kidneys and play protective roles in the repair of ischemic AKI and in renal ischemic preconditioning (Gandolfo et al., 2009; Kinsey et al., 2009, 2010). Therefore, we propose that the beneficial effect of RvD1 on inflammatory regression may involve not only peripheral inflammatory cells but also Tregs. In this study, we sought to investigate whether RvD1 attenuates IRI-AKI via affecting Tregs. This study demonstrates for the first time that RvD1 alleviates IRI-AKI possibly by increasing Tregs percentages via the ALX/FPR2 pathway.

## Materials and Methods

### Mice, Procedures and Interventions

Eight-week-old male C57BL/6 mice were purchased from Slake Laboratory Animal Company, Shanghai, China. All mice were fed a standard laboratory diet, provided unlimited access to drinking water, and housed in 50% humidity at room temperature on a 12 h/12 h light/dark cycle. Animal care was performed according to criteria established by the Animal Care Committee of Qingdao University. The IRI-AKI model was established by clamping the mouse bilateral renal pedicle for 60 min. In the sham operation group, a similar procedure was used except for clamping of the renal pedicle. RvD1 (Cayman Chemical, 5 μg/kg/d) or vehicle was administered through the tail vein at 30 min, 24 and 48 h after reperfusion. The dosage of RvD1 was selected based on previous reports (Kinsey et al., 2009). In some cases, RvD1 was given together with Boc-1 (a specific antagonist of ALX, China Peptide Co., 5 mg/kg/d). Blood, the kidneys, and spleen specimens were collected at the designated time points for further analysis. PC61 (BioLegend, San Diego, CA, United States, 100 μg), an anti-CD25 antibody, was used to deplete Tregs *in vivo* and was administered to the mice via the tail vein after reperfusion (Pasare and Medzhitov, 2004; Hu et al., 2013). Animals in the control group were given rat IgG (BioLegend, CA, United States).

### Histology

All mice were sacrificed at selected intervals. The kidneys were sequentially harvested, fixed with 4% paraformaldehyde, dehydrated and paraffin embedded. The paraffin tissue was sliced into 3 mm sections and stained with periodate acid-Schiff (PAS) for histological analysis. The histological evaluation was performed by grading tubular necrosis, cast formation, tubular dilation, and the loss of the brush border in a blinded manner to determine acute tubular necrosis (ATN) scores. Ten non-overlapping fields (400×) were randomly selected and scored as follows: 0 = no injury; 1 = less than 10%; 2 = 11% to 25%; 3 = 26% to 45%; 4 = 46% to 75%; and 5 = more than 76%.

### Biochemical Analysis

Blood samples were collected at 24 and 72 h and analyzed with a serum creatinine (Scr) kit (BioAssay Systems, Hayward, CA, United States).

### Single-Cell Suspensions From the Spleen and Kidneys

Single-cell suspensions of splenocytes and kidney cells were harvested from C57BL/6 mice as described previously (Zhang et al., 2014). Briefly, the spleen was finely minced with PBS, sequentially passed through a 200-mesh sieve and lysed with a red blood cell lysis buffer (BioLegend, San Diego, CA, United States). Kidney suspensions were additionally incubated with collagenase I (Sigma-Aldrich, 1.6 mg/ml) and DNase I (Sigma-Aldrich, 200 μg/ml) in RPMI-1640 medium (HyClone, Logan, UT, United States) at 37°C for 30 min. Then, cells were successively filtered through 70 and 40 μm mesh successively, and lysed with red blood cell lysis buffers.

### CD4^+^ T Cell Isolation and Intervention

Single-cell suspensions of splenocytes were harvested as described above. According to the manufacturer’s instructions, CD4^+^ T cells were purified with an EasySep Mouse CD4^+^ T cell enrichment kit (STEMCELL Technologies, Canada), and the purity was confirmed to be greater than 90% confirmed by FACS (Zhang et al., 2014). The purified naïve CD4^+^ T cells were cultured in RPMI-1640 medium supplemented with 10% fetal bovine serum (HyClone) at a density of 1 × 10^5^ in a 5% CO_2_ humidified incubator at 37°C. In addition, CD4^+^ T cells were induced by incubation with an anti-CD3 antibody (2.5 μg/ml, Invitrogen), an anti-CD28 antibody (5 μg/ml, Invitrogen), IL-2 (20 U/ml, Miltenyi Biotec) and TGF-β (2 ng/ml, Miltenyi Biotec) in the presence or absence of 10 nM RvD1 for 5 days (Chiurchiu et al., 2016). Cultures were supplemented with RvD1 every other day. After 5 days, cells were collected for FACS and real-time PCR analyses. In some cases, the purified naïve CD4^+^ T cells were preincubated with anti-GPR32 neutralizing antibodies (2 μg/ml, GeneTex) and/or anti-ALX/FPR2 neutralizing antibodies (2 μg/ml, Genovac) for 30 min before the incubation with RvD1 or vehicle and then stimulated with anti-CD3/CD28, IL-2, and TGF-β.

### Flow Cytometry

First, cells were incubated with Cytofix/Cytoperm (BioLegend) to permeabilize the cell membranes for 20 min at 4°C. Then, cells surfaces were stained with FITC-conjugated anti-CD4 and PE-conjugated anti-Foxp3 antibodies (eBiosciences, CA) according to the instructions. Finally, cytometry was performed with the BD FACS Calibur System (BD Bioscience). The plots were gated for CD4^+^ lymphocytes, and Tregs were identified as CD4^+^Foxp3^+^ T cells.

### Real-Time PCR

Total RNA was extracted with Trizol reagent, and cDNA was obtained by reverse transcription of 1 μg RNA according to the manufacturer’s instructions. Foxp3, KIM-1, Nephrin and β-actin were amplified by real-time fluorescence quantitative PCR kits (Takara Corporation, Japan) using SYBR Green master mix (Finnzyme, New England Biolabs). Relative mRNA levels were calculated by the 2^–ΔΔCt^ method and normalized to those of β-actin. The sequences of primers used for quantitative reverse transcription-polymerase chain reaction (RT-PCR) are listed in Table 1.

**TABLE 1:** The sequences of primers used for RT-PCR.

**Gene**	**Sense (5′→3′)**	**Antisense (5′→3′)**
Foxp3	GCACAAGTGCTTTGTGCGA GT	TGTCTGTGGTTGCAGACGTTGT
KIM-1	ACATATCGTGGAATCACAACGAC	ACTGCTCTTCTGATAGGTGACA
Nephrin	CAGGGAAGACAGCAACAAACAA	CAGGTTTTCAGATAGAGCCCAGA
β-actin	CTGAGAGGGAAATCGTGCGT	CCACAGGATTCCATACCCAAGA

### Cytokines Enzyme-Linked Immunosorbent Assay (ELISA)

The concentrations of IFN-γ, IL-10, IL-6, and TNF-α in blood samples were determined by ELISA kits (eBioscience) according to the manufacturer’s instructions. The absorbance of the final reactant was quantified at 450 nm with an ELISA plate reader (BioTek).

### Statistical Analysis

Values are expressed as the means ± SDs and represented by at least three independent experiments. A least significant difference (LSD) *t*-test or a one-way analysis of variance (ANOVA) was performed to compare differences among diverse groups using SPSS 13.0 software. Significance levels were set at *P* < 0.05 for all data analyses.

## Results

### RvD1 Alleviated Renal Injury in IRI-AKI

**FIGURE 1:**
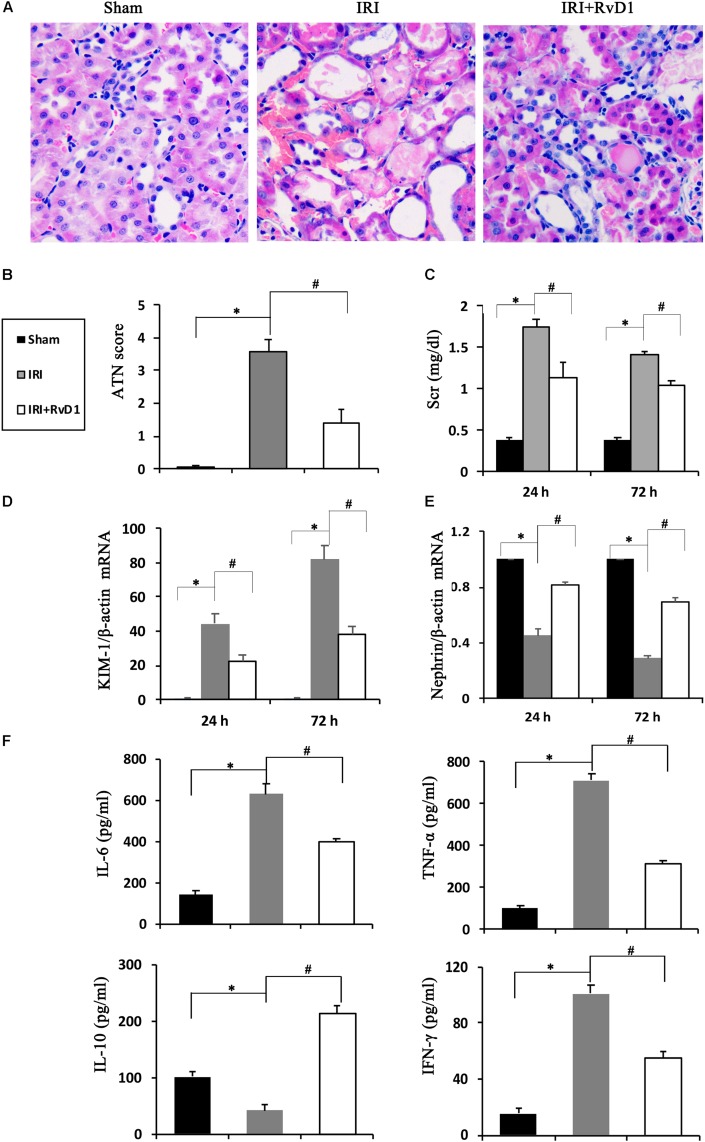
RvD1 protected against renal injury in IRI-AKI mice. The IRI-AKI model was established by clamping the bilateral renal pedicles for 60 min. RvD1 (5 μg/kg/d) or vehicle was administered to the mice via the tail vein at 30 min, 24 and 48 h after reperfusion. **(A)** Kidneys were stained by PAS (original magnification, 400×). **(B)** ATN scores at 72 h after reperfusion. **(C)** Serum creatinine levels at 24 and 72 h after reperfusion. The relative mRNA expression of KIM-1 **(D)** and Nephrin **(E)** at 72 h after reperfusion. **(F)** Serum IL-6, TNF-α, IL-10, and IFN-γ levels at 72 h after reperfusion as determined by ELISA. Values are expressed as the means ± SDs, *n* = 6–8 per group. ^∗^*P* < 0.05 versus sham; ^#^*P* < 0.05 versus IRI.

Duffield et al. (2006) demonstrated that IRI-AKI could result in the biosynthesis and release of RvD and protectins. To investigate the efficacy of RvD1 in IRI-AKI, mice were subjected to bilateral renal ischemia for 60 min. RvD1 was applied to the IRI mice via tail vein injection after reperfusion. The morphology and ultrastructure of kidney cells were nearly intact in the sham group, while IRI of the kidney resulted in protein cast formation, tubular epithelial cell sloughing, loss of the brush border, tubule dilation and infiltration of multiple inflammatory cells after 72 h. However, RvD1 administration significantly protected against tubule injury induced by IRI (Figure 1A). The semiquantitative assessment of ATN showed a lower score in the RvD1 group than in the IRI group (Figure 1B). In addition to the benefits to structural damage, RvD1 improved renal functions, which included reduced Scr levels at 24 and 72 h after reperfusion (Figure 1C). In addition, the mRNA level of kidney injury molecule-1 (KIM-1), a sensitive and specific biomarker for the early prediction of renal tubule injury (Vaidya et al., 2010), was increased at 24 and 72 h after reperfusion. As expected, RvD1 reduced the KIM-1 mRNA level compared with that in the IRI group (Figure 1D). Nephrin, a structural protein, plays an important role in maintaining the glomerular filtration barrier of the podocyte slit diaphragm (Kawachi et al., 1995; Ruotsalainen et al., 1999). It serves as an early marker of podocyte injury, and its reduced levels are largely associated with the loss of podocyte mass (Aaltonen et al., 2001). In our study, the nephrin mRNA levels were reduced at 24 and 72 h after reperfusion compared with those in the sham group; however, higher nephrin mRNA levels were observed in the RvD1-treated group (Figure 1E). Moreover, RvD1 administration reduced the levels of the proinflammatory cytokines IFN-γ, IL-6, and TNF-α but increased the level of the anti-inflammatory cytokine IL-10 in the serum (Figure 1F). In summary, these results suggested that RvD1 can alleviate renal lesions and protect renal function in IRI-AKI.

### RvD1 Increased the Percentages of Tregs in IRI-AKI

**FIGURE 2:**
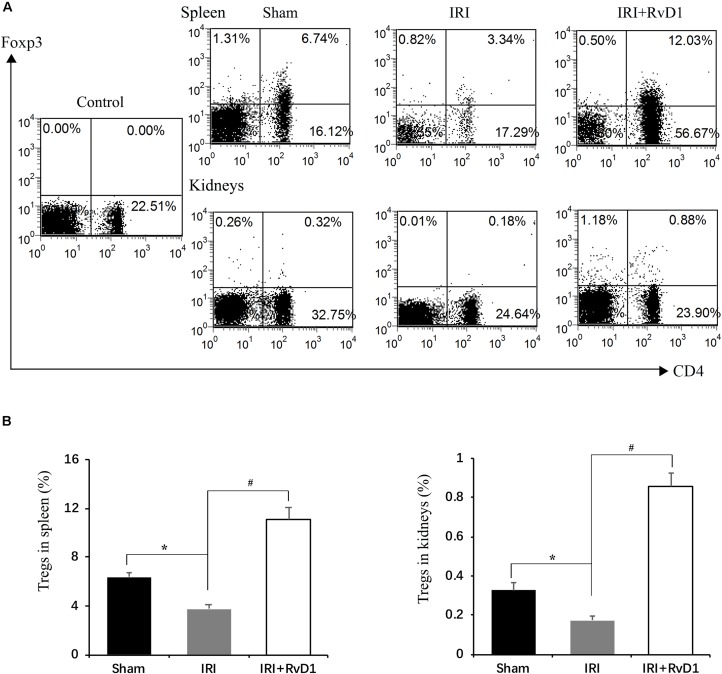
RvD1 increased the percentages of Tregs in IRI-AKI mice. At 72 h after reperfusion, single-cell suspensions were harvested from splenocytes and kidney cells. **(A)** Representative flow cytometry analysis of CD4^+^Foxp3^+^T cells obtained from the spleen or kidneys in IRI-AKI mice. The plots are gated for CD4^+^lymphocytes. The data are representative of 3 independent experiments. **(B)** Percentages of CD4^+^Foxp3^+^T cells in the spleen and kidneys of IRI-AKI mice. Values are expressed as the means ± SDs, *n* = 6–8 per group. *P < 0.05 versus sham; ^#^*P* < 0.05 versus IRI.

Foxp3^+^ Tregs inhibit innate and adaptive immune responses, which play important roles in ischemic preconditioning and ischemic AKI repair (Gandolfo et al., 2009; Kinsey et al., 2010). Therefore, we tested whether RvD1 administration could upregulate the proportions of Tregs in IRI-AKI mice. Single cells isolated from the spleen were detected by FACS to assess CD4^+^Foxp3^+^ T cell percentages. We found that RvD1 increased Treg percentages in the spleen after 72 h of continuous administration (Figures 2A,B). Additionally, the Treg percentages in the kidneys increased after RvD1 administration (Figures 2A,B), although a small number of Tregs were observed in the kidneys of the IRI-AKI mice. Therefore, these results indicated that RvD1 enhances the Treg percentages in both the kidneys and spleen in IRI-AKI mice.

### Depletion of Tregs Reversed the Beneficial Effects of RvD1 on IRI-AKI

**FIGURE 3:**
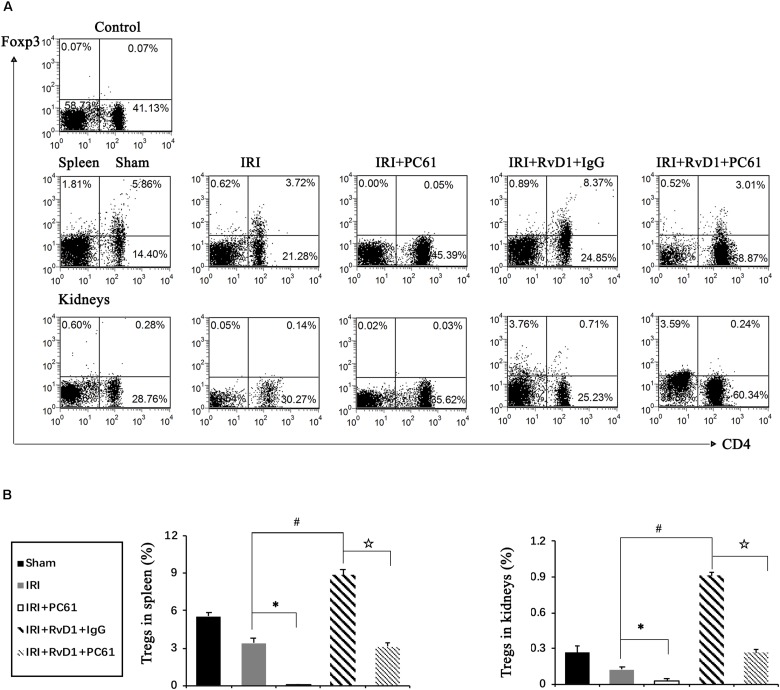
PC61 administration reduced Treg percentages in the spleen and kidneys of IRI-AKI mice. The IRI-AKI model was established, and PC61 or rat IgG was administered via the tail vein after RvD1 or vehicle interventions. Single-cell suspensions were generated from the spleen and kidneys at 72 h after reperfusion. **(A)** Representative flow cytometry analysis of CD4^+^Foxp3^+^ T cells obtained from the spleen or kidneys of IRI-AKI mice treated with or without RvD1, PC61 or rat IgG. The plots are gated for CD4^+^lymphocytes. The data are representative of 3 independent experiments. **(B)** The percentage of CD4^+^Foxp3^+^ T cells in the spleen or kidneys. Values are expressed as the means ± SDs, *n* = 6–8 per group. ^∗^*P* < 0.05, IRI + PC61 versus IRI; ^#^*P* < 0.05, IRI + RvD1 + IgG versus IRI; ✩ *P* < 0.05, IRI + RvD1 + IgG versus IRI + RvD1 + PC61.

**FIGURE 4:**
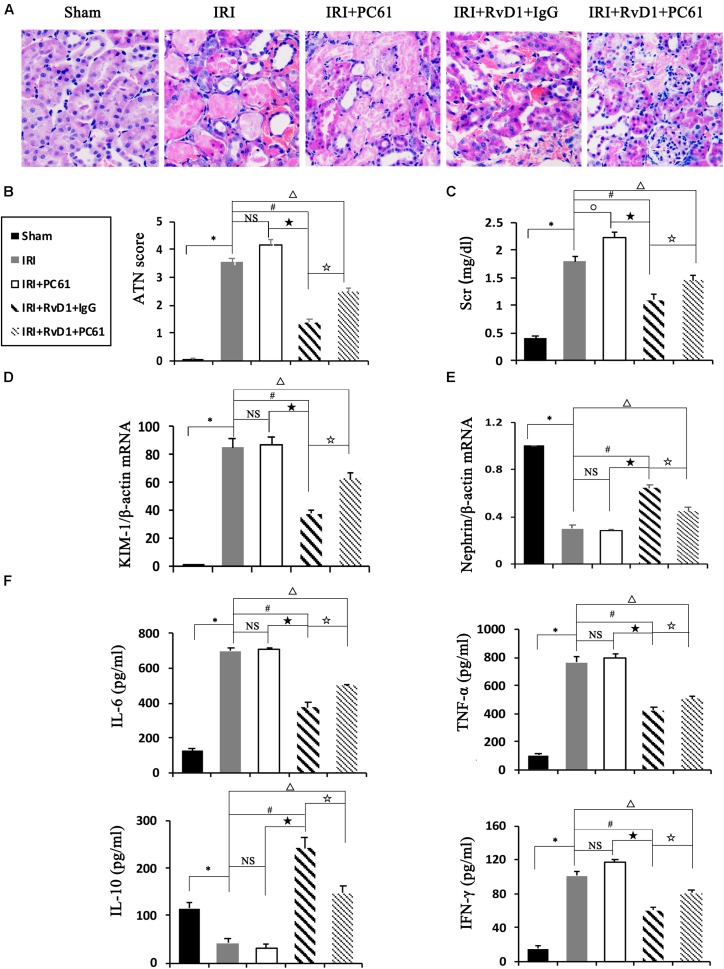
PC61 reversed the beneficial effects of RvD1 on IRI-AKI. **(A)** Kidneys were stained by PAS (original magnification, 400×). **(B)** ATN scores at 72 h after reperfusion. **(C)** Serum creatinine levels at 72 h after reperfusion. The relative mRNA expression of KIM-1 **(D)** and Nephrin **(E)** at 72 h after reperfusion. **(F)** Serum IL-6, TNF-α, IL-10 and IFN-γ levels at 72 h after reperfusion as determined by ELISA. Values are expressed as the means ± SDs, *n* = 6–8 per group. ^∗^*P* < 0.05, IRI versus Sham; ^#^*P* < 0.05, IRI + RvD1 + IgG versus IRI; ★ *P* < 0.05, IRI + PC61 versus IRI + RvD1 + IgG; ✩ *P* < 0.05, IRI + RvD1 + IgG versus IRI + RvD1 + PC61; ^Δ^*P* < 0.05, IRI + RvD1 + PC61 versus IRI; °*P* < 0.05, IRI + PC61 versus IRI; NS: not significant.

To further define the role of Tregs in the beneficial effects of RvD1 on IRI-AKI, Tregs were depleted *in vivo*. PC61 (an anti-CD25 antibody, 100 μg) can reportedly effectively remove Tregs *in vivo* without affecting non-Tregs (Pasare and Medzhitov, 2004; Hu et al., 2013). After PC61 administration, the percentages of Tregs in the spleen and kidneys were significantly reduced in IRI-AKI mice (Figures 3A,B), which indicated that the depletion of Tregs was successful. In the IRI-AKI mice administered RvD1, PC61 reversed the beneficial effects of RvD1 on IRI-AKI, which included aggravated tubular injury (Figure 4A), increased ATN scores (Figure 4B) and renal function deterioration (Figure 4C). Moreover, higher KIM-1 mRNA levels (Figure 4D) and lower nephrin mRNA levels (Figure 4E) were observed in the PC61 group than in the rat IgG group. In addition, compared with rat IgG, PC61 treatment increased the levels of the proinflammatory cytokines IFN-γ, IL-6, and TNF-α but decreased the level of the anti-inflammatory cytokine IL-10 (Figure 4F). These experimental results demonstrate that the protection of RvD1 in IRI-AKI is related to the increased percentage of Tregs.

### RvD1 Induced the Generation of Induced Tregs (iTregs) via ALX/FPR2 Receptors

**FIGURE 5:**
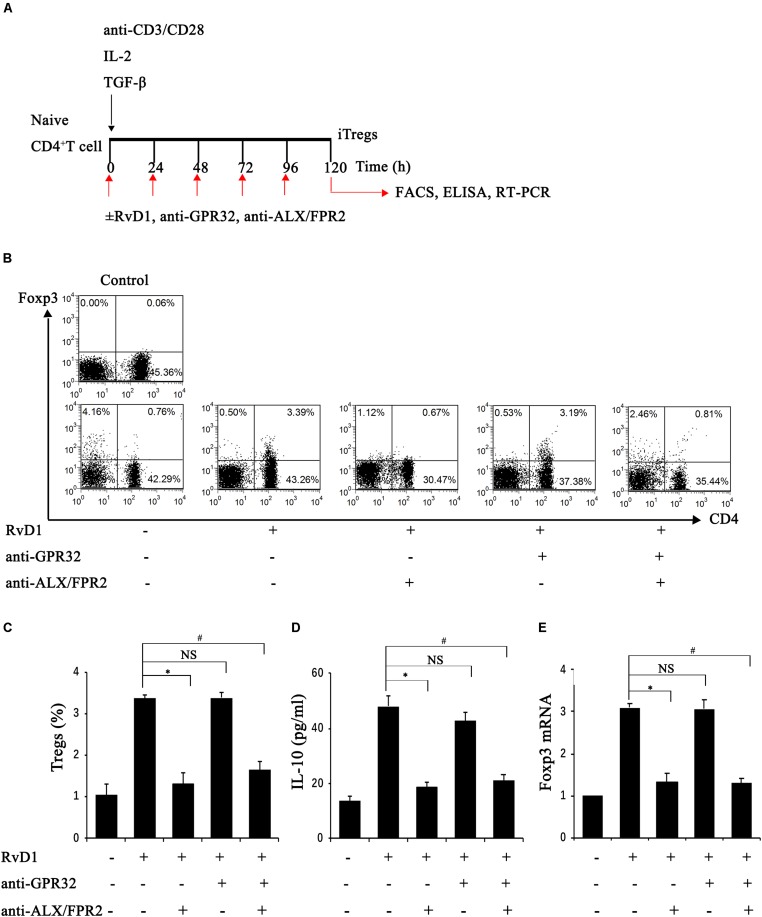
RvD1 induced the generation of iTregs via the receptor ALX/FPR2. **(A)** The sketch map of iTreg generation. **(B)** Representative flow cytometry analysis of iTregs generated with or without RvD1, an anti-GPR32 neutralizing antibody, or an anti-ALX/FPR2 neutralizing antibody at 96 h after incubation under the condition of iTreg generation. The plots are gated for CD4^+^ lymphocytes. The data are representative of 3 independent experiments. **(C)** The percentage of iTregs. **(D)** IL-10 levels in the supernatants of iTregs as determined by ELISA. **(E)** The mRNA expression of Foxp3 in iTregs as determined by RT-PCR. Values are expressed as the means ± SDs, *n* = 6 per group. ^∗^*P* < 0.05, RvD1 versus RvD1 + anti-ALX/FPR2 antibody; ^#^*P* < 0.05, RvD1 versus RvD1 + anti-ALX/FPR2 antibody + anti-GPR32 antibody; NS: not significant, RvD1 versus RvD1 + anti-GPR32 antibody.

Further, we conducted another study to explore whether RvD1 could affect the generation of iTregs, which develop from naïve CD4^+^ T cells under antigen and TGF-β stimulation (Chiurchiu et al., 2016). To this end, naïve CD4^+^ T cells were stimulated with RvD1 or vehicle under Treg-inducing conditions *in vitro* (Figure 5A). At 96 h, the percentages of CD4^+^Foxp3^+^ Tregs (Figures 5B,C), Foxp3 mRNA expression (Figure 5E), and serum IL-10 levels (Figure 5D) were all increased in the presence of RvD1 compared with the control vehicle. These results suggest that RvD1 affects not only the induction of Tregs but also their specific functional properties.

To verify the potential molecular mechanism of RvD1 in iTreg regulation, we assessed the role of RvD1 receptors in iTregs. GPR32 and ALX/FPR2 are known receptors of RvD (Krishnamoorthy et al., 2010; Norling et al., 2012) and because Chiurchiu et al. demonstrated that iTregs express both GPR32 and ALX/FPR2 (Chiurchiu et al., 2016), we focused on these receptors. Interestingly, preincubation with anti-ALX/FPR2 neutralizing antibodies alone or in combination with anti-GPR32 neutralizing antibodies abrogated the enhancement effect of RvD1 on iTregs, while anti-GPR32 neutralizing antibodies alone did not (Figures 5B–E). This result suggests that the receptor ALX/FPR2 mediates the effects of RvD1 on iTregs *in vitro*.

### Boc-1 Reversed the Protective Effect of RvD1 on IRI-AKI

**FIGURE 6:**
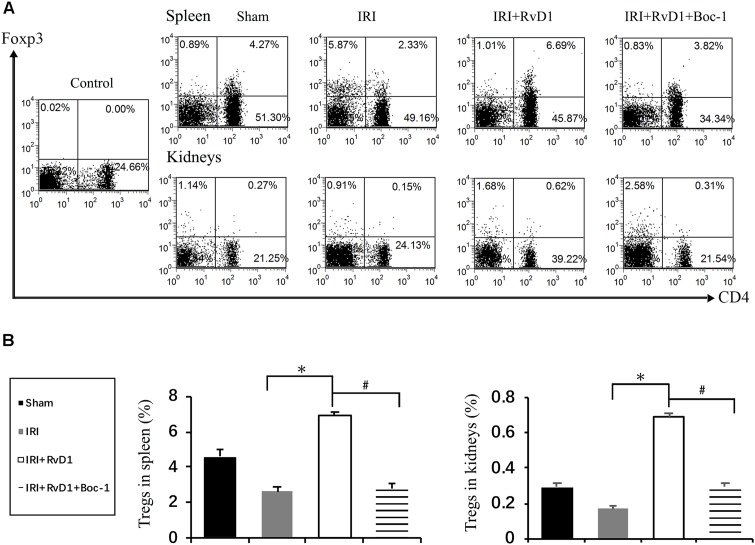
Boc-1 abrogated the effect of RvD1 on the spleen and kidney cell Treg percentages. The IRI-AKI model was established and Boc-1 was administered via the tail vein after RvD1 or vehicle interventions. **(A)** Representative flow cytometry analysis of CD4^+^Foxp3^+^ T cells obtained from the spleen or kidneys of IRI-AKI mice treated with or without RvD1 or Boc-1. The plots are gated for CD4^+^ lymphocytes. The data are representative of 3 independent experiments. **(B)** The percentage of CD4^+^Foxp3^+^ T cells in the spleen or kidneys. Values are expressed as the means ± SDs, *n* = 6–8 per group. ^∗^*P* < 0.05, IRI versus IRI + RvD1; ^#^*P* < 0.05, IRI + RvD1 versus IRI + RvD1 + Boc-1.

**FIGURE 7:**
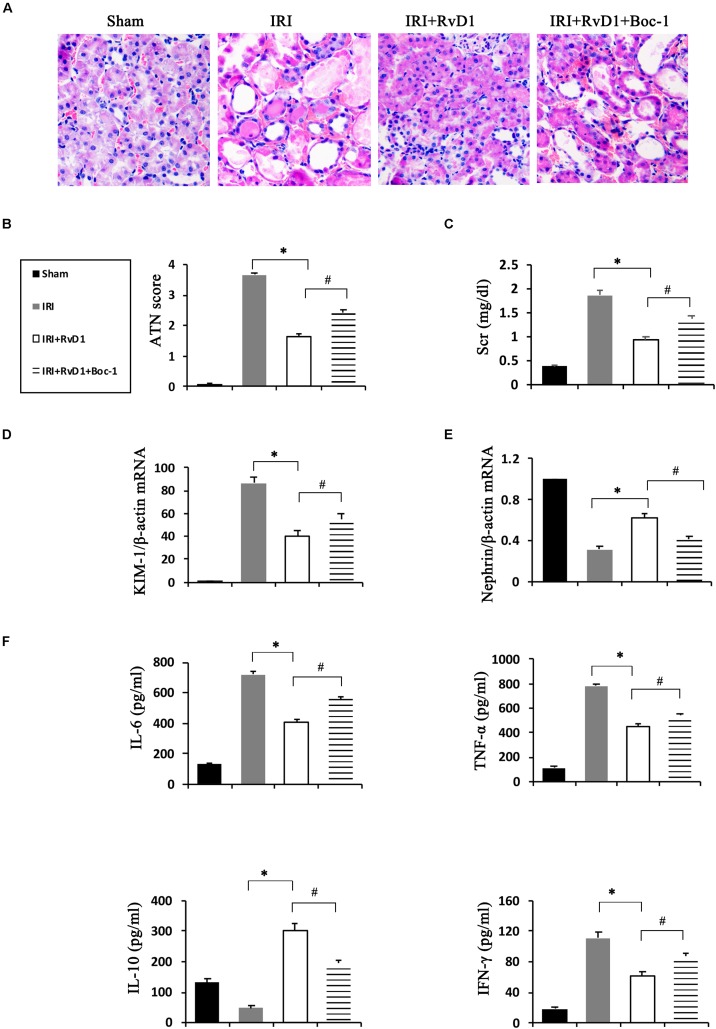
Boc-1 reversed the improved therapeutic efficacy of RvD1 in IRI-AKI. **(A)** Kidneys were stained by PAS (original magnification, 400×). **(B)** ATN scores at 72 h after reperfusion. **(C)** Serum creatinine levels at 72 h after reperfusion. The relative mRNA expression of KIM-1 **(D)** and Nephrin **(E)** at 72 h after reperfusion. **(F)** Serum IL-6, TNF-α, IL-10 and IFN-γ levels at 72 h after reperfusion as determined by ELISA. Values are expressed as the means ± SDs, *n* = 6–8 per group. ^∗^*P* < 0.05, IRI versus IRI + RvD1; ^#^*P* < 0.05, IRI + RvD1 versus IRI + RvD1 + Boc-1.

Boc-1 is a selective RvD1-receptor ALX/FPR2 antagonist. To further verify that RvD1 increases the percentage of Tregs through the ALX/FPR2 pathway *in vivo*, Boc-1 was administered to IRI-AKI mice. We found that Boc-1 reduced the Treg percentages in the spleen and kidneys of IRI-AKI mice treated with RvD1 (Figure 6). Additionally, compared with RvD1 treatment, the administration of Boc-1 *in vivo* led to pathological changes (widespread protein cast, tubular necrosis and inflammatory cell infiltration; Figure 7A), increased in ATN scores (Figure 7B), renal dysfunction (manifested as high levels of Scr; Figure 7C) and inflammatory cytokines infiltration (high serum levels of IFN-γ, IL-6 and TNF-α; Figure 7F). Moreover, Boc-1 treatment increased the mRNA levels of KIM-1 (Figure 7D) but reduced those of Nephrin (Figure 7E) compared to those in the RvD1 treatment group. This result demonstrated that Boc-1 reversed the protective effect of RvD1 on IRI-AKI.

## Discussion

RvD1, one of the most extensively studied resolvins, can promote the resolution of inflammation by inhibiting inflammatory cell infiltration, downregulating cytokine secretion and promoting neutrophil apoptosis (Schwab et al., 2007). It is reported to play protective roles in a variety of disease models, including acute lung injury, peritonitis, wound infection, insulin resistance and atherosclerosis models (Bento et al., 2011; Weylandt et al., 2012). In the field of nephrology, RvD1 can also preserve renal function and inhibit fibrosis in multiple kidney diseases, such as obstructive nephropathy (Qu et al., 2012), adriamycin-induced AKI (Zhang et al., 2013), lipopolysaccharide (LPS)-induced AKI (Chen et al., 2014), paraquat-induced AKI (Hu et al., 2019), and IRI-AKI (Duffield et al., 2006). In our research, RvD1 administration alleviated renal injury and protected renal function in IRI-AKI, results that were identical to those of previous studies.

In addition to limiting inflammation, RvD1 also plays an important role in adaptive immune mediation. Chiurchiu et al. reported that RvD1 could promote the generation of Foxp3^+^ Tregs (Chiurchiu et al., 2016). Luo et al. suggested that RvD1 could increase Treg activity and the macrophage phagocytosis of apoptotic T cells, which was shown to contribute to disease recovery in rats with experimental autoimmune neuritis (Luo et al., 2016). Tregs are lymphocytes with immunosuppressive properties that are commonly identified by their expression of CD4 and CD25 on the cell surface and upregulated levels of the transcription factor Foxp3 (Fontenot et al., 2003). With the development of a tissue digestion and sieving technique followed by flow cytometry, Tregs were discovered in the normal kidney (Ascon et al., 2006). Despite less infiltration in the normal kidney (less than 1% as assessed by FACS) (Gandolfo et al., 2009; Kinsey et al., 2009), Tregs still play an important role in many kidney diseases, such as AKI and progression to CKD. Tregs suppress innate immunity and participate in the repair of ischemic AKI and in renal ischemic preconditioning (Gandolfo et al., 2009; Kinsey et al., 2009, 2010). Our studies demonstrated that RvD1 administration could increase the Treg percentages in the spleen and kidneys of IRI-AKI mice. However, whether the increased percentages of Tregs were important for the protective effect of RvD1 remained unknown. To better understand the causal relationship between the induction of Treg activity by RvD1 and its renal protective effect, PC61, an anti-CD25 antibody, was used to deplete Tregs, which mitigated the effect of RvD1 on ischemic injury. These results suggest that the increased percentages of Tregs induced by RvD1 treatment may contribute to the beneficial effects of RvD1 on IRI-AKI.

Treg depletion experiments are complex. The administration of PC61 to IRI-AKI mice slightly increased the average ATN score and the inflammatory cytokine levels compared with those in IRI group, but the differences were not statistically significant. This result was consistent with those reported by Bai et al. (2018) and Gandolfo et al. (2009). However, Gandolfo reported that PC61 administration reduced Scr levels within 1 day, increased tubular damage in the outer medulla after 3 days and persistently increased tubular damage after 10 days in IRI-AKI mice, while Treg transfer was associated with histological changes only at 10 days. These results suggested that Treg depletion and transfer require some time to become effective, which may be related to the lower number of Tregs in the kidney. These studies may further explain our results, and we hypothesized that as the IRI time extends in mice, the effect of PC61 on renal pathology will gradually appear. In addition, the treatment of Tregs-depleted mice with RvD1 had some protective effects in histopathology, renal function and inflammatory cytokines levels compared with those in the IRI group. Therefore, we speculate that RvD1 protects IRI-AKI mice by increasing the numbers of not only Tregs but also other cells, such as TH1 and Th17 cells.

The mechanism of Treg amplification in mice treated with RvD1 may be associated with the proliferation of preexisting Tregs or the transformation of naïve CD4^+^ T cells. Recently, Chiurchiu et al. reported that RvD1 could enhance the *de novo* generation of Foxp3^+^ Tregs and further confirmed the conclusion drawn *in vivo* regarding Elovl2^–/–^ mice, which are deficient for elongase 2, the key enzyme involved in the synthesis of DHA (the precursor of RvD) (Chiurchiu et al., 2016). *In vitro*, we further evaluated whether RvD1 could affect the transformation of non-Tregs to Tregs. iTregs develop from naïve CD4^+^ T cells under stimulation by antigen and TGF-β (Yamagiwa et al., 2001). Therefore, purified naïve CD4^+^ T cells were incubated with RvD1 under the condition of Treg induction. Our data showed that RvD1 could potentiate iTreg differentiation, with significantly higher Foxp3 mRNA expression levels compared to those in the control group. The GPR32 and ALX/FPR2 are two receptors that have been shown to transmit RvD1 signals, but only ALX/FPR2 has been identified in rodents (Recchiuti, 2013). Interestingly, after the addition of an anti-ALX/FPR2 neutralizing antibody, the Treg percentage, Foxp3 mRNA level and IL-10 level were reduced. However, this effect was not observed after the additional application of an anti-GPR32 neutralizing antibody. Furthermore, *in vivo* studies showed that the administration of Boc-1, a selective RvD1-receptor ALX/FPR2 antagonist, reduced the percentages of Tregs in the spleen and kidneys of IRI-AKI mice treated with RvD1 and reversed the beneficial effects of RvD1 on tubular injury. Therefore, these results suggest that RvD1 enhances the generation of iTregs and the protective efficacy of RvD1 against IRI-AKI via the ALX/FPR2 pathway.

In summary, our studies demonstrated that RvD1 administration improved renal injury in IRI-AKI. This amelioration efficacy was associated with an increased percentage of Tregs induced by the ALX/FPR2 pathway.

## Data Availability Statement

The raw data supporting the conclusion of this article will be made available by the authors, without undue reservation, to any qualified researcher.

## Ethics Statement

The animal study was reviewed and approved by the Animal Care Committee of Qingdao University.

## Author Contributions

HL and YX designed the study and wrote the manuscript. CW and LZ contributed to the original data. SS performed the pathological image analysis. LL and BZ contributed to the flow cytometry analysis. XS performed the cell experiments. JS performed the ELISA and RNA data analysis.

## Conflict of Interest

The authors declare that the research was conducted in the absence of any commercial or financial relationships that could be construed as a potential conflict of interest.
